# Application of machine learning methods for predicting infant mortality in Rwanda: analysis of Rwanda demographic health survey 2014–15 dataset

**DOI:** 10.1186/s12884-022-04699-8

**Published:** 2022-05-04

**Authors:** Emmanuel Mfateneza, Pierre Claver Rutayisire, Emmanuel Biracyaza, Sanctus Musafiri, Willy Gasafari Mpabuka

**Affiliations:** 1 African Centre of Excellence in Data Science, University of Rwanda, Kigali, Rwanda; 2 Applied Statistics Department, University of Rwanda, Kigali, Rwanda; 3 Prison Fellowship Rwanda, Kigali, Rwanda; 4 Clinical Department of Internal Medicine, University of Rwanda, Kigali, Rwanda; 5 Transparency International Rwanda, Kigali, Rwanda

**Keywords:** Infant mortality, Machine Learning, Logistic regression, Model accuracy

## Abstract

**Background:**

Extensive research on infant mortality (IM) exists in developing countries; however, most of the methods applied thus far relied on conventional regression analyses with limited prediction capability. Advanced of Machine Learning (AML) methods provide accurate prediction of IM; however, there is no study conducted using ML methods in Rwanda. This study, therefore, applied Machine Learning Methods for predicting infant mortality in Rwanda.

**Methods:**

A cross-sectional study design was conducted using the 2014–15 Rwanda Demographic and Health Survey. Python software version 3.8 was employed to test and apply ML methods through Random Forest (RF), Decision Tree, Support Vector Machine and Logistic regression. STATA version 13 was used for analysing conventional methods. Evaluation metrics methods specifically confusion matrix, accuracy, precision, recall, F1 score, and Area under the Receiver Operating Characteristics (AUROC) were used to evaluate the performance of predictive models.

**Results:**

Ability of prediction was between 68.6% and 61.5% for AML. We preferred with the RF model (61.5%) presenting the best performance. The RF model was the best predictive model of IM with accuracy (84.3%), recall (91.3%), precision (80.3%), F1 score (85.5%), and AUROC (84.2%); followed by decision tree model with model accuracy (83%), recall (91%), precision (79%), F1 score (84.67%) and AUROC(82.9%), followed by support vector machine with model accuracy (68.6%), recall (74.9%), precision(67%), F1 score (70.73%) and AUROC (68.6%) and last was a logistic regression with the low accuracy of prediction (61.5%), recall (61.1%), precision (62.2%), F1 score (61.6%) and AUROC (61.5%) compared to other predictive models. Our predictive models showed that marital status, children ever born, birth order and wealth index are the 4 top predictors of IM.

**Conclusions:**

In developing a predictive model, ML methods are used to classify certain hidden information that could not be detected by traditional statistical methods. Random Forest was classified as the best classifier to be used for the predictive models of IM.

## Background

The infant mortality rate (IMR) is regarded as an important national health indicator endangering health of both mother and new-born because it is particularly sensitive to structural factors such as socioeconomic development and basic living conditions [1]. From 1990 to 2017, the IMR in Sub-Saharan Africa (SSA) countries fell from 182 to 58 deaths per 1000 live births [2]. It is still high when compared to the World's Sustainable Development Goal (SDG-3) number 3, which seeks to "ensure healthy lives and promote the well-being of all at all ages" by 2030, with the main objective of reducing a high prevalence of neonatal deaths to at least 12 live births and under-five mortality to at least 25 live births [3, 4]. Although this infant mortality remains a public health burden particularly in developing countries, there is a significant progress in child health due to efforts and strategies taken for combating risk factors of this health burden [4]. Recent studies indicated that an automatic intelligent system based on artificial intelligence (AI), machine learning (ML) and deep learning (DL) have the potential to accelerate this progress [5] and these methods have demonstrated the significant performance in various fields including public health and medicine domains [4, 6–8]. These methods are followed by computational intelligence in this progress [9].

Preceding researchers used a variety of models and methodologies to investigate risk factors for IM and factors associated with infant mortality was found using conventional methods including discrete-time logistic log-likelihood models, survival analysis, and multivariate decomposition [10, 11]. However, the studies on AI stated that the ML are better methods than conventional methods to provide prediction of epidemiological concerns when applied on a large sample size, almost epidemiological studies on these methods are from developed countries [3, 4, 12], only few studies conducted in SSA [13]. The research in SSA failed to fully explain the reasons that account for heterogeneity in child mortality and this failure is due to using traditional methods or applying the machine learning on small sample size of the population [14–17]. ML approaches are employed to represent a unique logical and critical ways to unravel preceding tendencies that were not found in previous studies [18]. These methods build on prevailing statistical methods by employing procedures that have not been established and predicated on a priori assumptions about the distribution of data [19, 20]. Additionally, Advanced Machine Learning (AML) scientifically and importantly to provide predictions infant mortality and has the ability to predict perinatal or neonatal mortality. Indeed, the policy makers may use the results in designing the appropriate health interventions to reduce risk factors health issue [18, 21, 22]. Additionally, AML systems are superior to classic analytical approaches such as multiple logistic regression models for accurately predicting factors of infant mortality [13, 20]. Additionally, ML approaches were undertaken in medical settings for prognostication and mortality prediction in this clinical environment; however, ML methods have yet to be used in community health studies where it could represent a potential transformative tool [4, 23, 24].

Moreover, previous studies that utilised ML approaches to predict infant mortality reported that Random Forest performed well with an accuracy that varies from 67.2% to 97.1%, followed by Logistic Regression model with 86.1%, and K-nearest neighbour with 85.6% [25, 26]. In addition to that, the ML models have potentials to perform better than the traditional statistical models because their ability to deal with non-linear complex data, multiple interactions between determinants and handle multiple factors and chain of events simultaneously [5, 27]. Also, ML is a prediction method that importantly determine not only who are a high risk to be died but also when the women and infants are at a higher risk [13, 28]. Comparing traditional models, ML methods can disentangle relations that are nonlinear or binary and can produce highly stable estimates or predictions in large datasets that are frequently challenging to find correlations [5, 27, 29].

ML is a branch of AI used in medical settings for performing multifaceted tasks that typical require human intelligence like predicting infant and neonatal deaths. The best overall model was linear discriminant analysis which has high number of features [4, 27]. Further, data mining techniques such as Decision tree, Random Forest, Support Vector Machine, and Nave Bayes algorithms, the prevalence of infant mortality and its associated determinants were conveyed [13]. They also predict babies and child mortality in different countries like Ethiopia where the prediction was conducted using the ML methodologies. Further, the random forest is a good classifier when compared to others, with 98.43% accuracy in imbalanced train data and 95.22% average accuracy in balanced train data [26]. Annual population growth is closely connected with child mortality, according to the decision tree. The model that was created has a high level of acceptability [13, 29]. Indeed, Naive Bayes classifier outperformed the other predictive classifiers, with an accuracy of 98.2% and a Receiver Operating Characteristic (ROC) Area of 0.92 [26, 30].

Moreover, the decision tree model performed better in terms of accuracy [26, 31]. Neural network and decision tree data mining techniques were used to predict the risk of child mortality, and the results showed that child mortality was linked to the environment, household literacy, household health, child age, availability of windows in the house, household water, and even household ethnicity [32]. In preceding research focused on predicting neonatal and perinatal mortality using ML approaches, researchers reduced the risk of bias and improve predictive accuracy [13]. In particular, controlling missing values and the selection of variables seems to create a significant impact in ML research. So, infants with missing information were excluded to reduce the risk of bias due to missing values [4]. Although these methods of prediction have better performance than conventional models when applied to large datasets given their ability to outline composite relationships and detect novel relationship between variables [3, 4], the studies on AML remain rare in SSA [14, 20, 33]. No study conducted in Rwanda using the AML methods for predicting any epidemiological concern like IM. Therefore, the present research aimed to apply AML for predicting infant mortality in Rwanda based on 2014–15 Rwanda Demographic and Health Survey (RDHS). We hypothesized that the AML have higher predictive accuracy than conventional logistic regression models in Rwanda.

### Conceptual framework

**Fig. 1:**
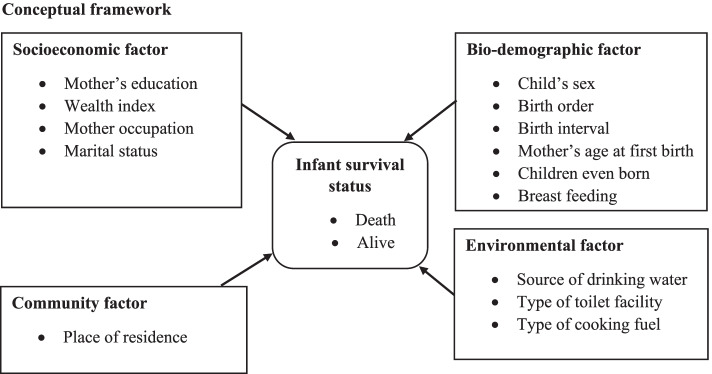
Conceptual framework

We adjusted the conceptual framework of IM based on the analytical framework documented in the research of Mosley and Chen [21]. The analytical framework of child mortality was first proposed by Mosley and Chen in 1984 and later adapted with an emphasis on variation in infant and child mortality explained by differentials in socio-economic, bio-demographic, and household environmental conditions, nutrients deficiency factors and community factors (Fig. 1).

## Methods

### Study design

This cross-sectional study design was carried out using the data from the 2014–15 Rwanda Demographic Health Survey which is a nationally representative household survey.

### Settings and participants

The study was carried in Rwanda which is the country of thousand hills. It is also a country situated at the East Africa where it is also one of the members of East African Community members. From the Indian Ocean there are 1200 km while there is around 200 km from the Atlantic Ocean. This country also has four provinces and Kigali City namely Eastern Province, Western Province, Northern Province, Southern Province and Kigali city which is the capital city of this country. Basically, this survey targeted the women who were 15–49 years old and all participants were enrolled from the RDHS that employed the samples households. This demographic health survey also enrolled the total of 30,058 households composed of 23,989 (80%) households from urban settings and 6,069 (20%) households that were selected from the urban settings. Each household among those that were selected was represented by one woman who was in the period of fertility. All participants were administered the Households Questionnaires. Each participant was asked to provide the details of birth history in the chronological orders. Hereby, the participants started with their first newborn and ended with their last newborn. Thus, a total of 492 clusters was employed to select the study participants. Within the RDHS, the multistage stratified sampling methods was also employed so that the researchers could get accurate information from the participants. This survey was conducted from 9^th^ November 2014 and ended at 8^th^ April 2015 [34].

### Study variables

The dependent variable for this study was infant mortality which was defined as the death of a live birth before the first birthday and it was estimated based on the information collected from the birth history section of the questionnaire which was administered to individual women. The independent variables included place of residence, marital status, maternal education, maternal occupation, wealth index, mother’s age at first birth, sex of a child, birth order, birth interval, children ever born, breastfeeding, source of drinking water, type of toilet facility and type of cook fuel. Those variables were selected using the existing literature review of infant mortality.

### Procedures and sample size

The total sample size of this study was 30,058 babies born to women aged 15 to 49 years old, based on the RDHS 2014–15 dataset of all births file (BR). This study was conducted among a total number of 30,058 babies. Among them 1952 babies were died while there were 28,106 babies alive. Among the overall the sample size, 29,233 were single births, 820 twins and 5 triplets babies.

The infant mortality rate, which is defined as the probability of a live-born child dying before reaching the age of one, is one of the most sensitive and often used indices of a population's social and economic progress. ML methods, as opposed to traditional methods, are typically flexible and nonparametric when it comes to producing predictions or classifications from data, and they employ algorithms to uncover patterns in data using variable and model selection procedures [35]. Using the RDHS 2014–15 dataset, a nationally representative cross-sectional survey, this work proposes the use of machine learning methods such as logistic regression, decision tree, random forest, and support vector machine to create infant mortality forecasting models in Rwanda. Because the dependent variable (infant mortality) has a binary answer (death/alive), these methods were applicable for this study. The dataset was divided into two parts: training and testing datasets, with the training dataset accounting for 80% of the total and the test dataset accounting for 20%. The performance of predictive models was assessed using confusion matrix, accuracy, precision, recall, and F1 score, as well as Area under Curve and AUROC. ML on the other hand, is strongly reliant on the quality and consistency of accessible data. To acquire the best results from any machine learning method, you must first prepare your data [7, 18]. Missing values were replaced with the median where possible to avoid any misleading information from the existing data, and a few variables with multiple missing values were excluded from this analysis. Furthermore, the random forest algorithm was utilized in this study to determine the optimum feature selection that contributes to new-born mortality, and the random oversampling approach was used to balance the distribution of the disproportionate categories of the dependent variable, infant mortality.

### Data analysis

An in-depth analysis was performed to examine the relationship of independent variables with infant mortality at 5% of statistical significance level and 95% confidence interval and, predictive modelling which takes into account ML algorithms were used to come up with objective predictions about infant mortality. Indeed, data processing is a data mining technique that transforms raw data into an understandable format. Raw data (real-world data) is always incomplete and that data cannot be sent through a model. Preparing data is required to get the best results what done within ML algorithms on data [18, 36]. Variables with many missing values were not considered in this study. Feature selection is a process of reducing the number of variables during developing a predictive model. The feature selection methods were used in data pre-processing to achieve efficient data reduction. A random forest algorithm was employed to identify the best features that contribute to infant mortality. Indeed, the data imbalanced is a special case for classification problem where the class distribution is not uniform among the classes. In this study, there was disproportion encountered in the outcome variables. The random oversampling method was used to balance the distribution of classes of infant mortality. Random oversampling involves supplementing the training data with multiple copies of some of the minority classes. In this study, random oversampling was applied to increase the number of infant mortalities in to balance with children alive. The study employed ML methods to find patterns and predict the risk of mortality. For comparing with the results from ML methods and traditional methods, we computed bivariate logistic regression analysis using a chi-square test for assessing significant variables of IM. Then, all significant variables were exported into multiple logistic regression models based on odds ratio. ML uses algorithms to discover patterns in data using variable and model selection methods. Python software version 3.8 was employed for statistical testing and computes ML methods through Logistic Regression, Random Forest, Decision Tree, and Support Vector Machine. We computed logistic regression analyses using STATA version 13. Statistical level of the significance level of 5% and the 95% of confidence intervals were considered.

## Ethics approval and consent to participate

The present study was exempt from searching the ethical approval because the RDHS 2014/15 was previously carried out under ethical approval from the Institutional Review Board (IRB) of the Rwanda National Ethics Committee (RNEC). Authors were authorized to use the dataset from DHS program that is publicly accessible on the NISR website. The RDHS was conducted in accordance of the guidelines and regulations stated in the Declaration Helsinki. The confidentiality and anonymity were ensured in the RDHS. In the prior approval, the women of reproductive age who were age 18–49 years provided oral and written informed consent forms to take part in the survey. In the cases on the minor participants (those women aged 15–17 years), illiterate and dead participants; the assent form was obtained from them while written informed consent were simultaneously provided by their guardians or parents who were adults.

## Results

### Descriptive results of the background characteristics

Around 6.5% of infants died before their first birthday out of the 30058 infants of the sample. The majority (79.8%) of infants were from the rural area. The majority 58.9% of infants born from married women, 20.7% born to women lived with their partners, 8.4% were born to divorced/separated women, 8.2% born to widowed women, and only 3.8% were born to single women. The majority 67.9% of infants were born to mothers who completed primary level, 22.5% were born to mothers who had no formal education, and 9.6% were born to mothers who have completed at least secondary level. Over 95.0% of infants born to mothers were employed while 5.0% of them were born to mothers who were unemployed. The wealth index difference among families which participated in the survey was 42% low, 20% middle, and 38.1% high. The majority 67.3% of the infants were born to mothers aged between 20 and 34 years compared to 32.5% and 0.2% of infants born respectively to mothers aged below 20-year-old and 35 years old and over. The majority (50.6%) of the infants were males. The majority (51.7%) of infants who were born to mothers who had 1 or 2 births followed by those who were born to mothers who had 3–4 births (28.6%). Most (52.3%) of the births occurred to mothers whose preceding birth interval was 24 months and above compared to 18.2% of births occurring to mothers with preceding birth intervals of less than 24 months. The majority of infants (42.2%) were born to mothers who had between 4 and 6 children. The majority (60.5%) of births were breastfed. The majority (60.7%) were from the families that had from un-unimproved sources of water. Over 95.3% of infants occurred in households with improved toilet facility while. More than 86% of infants were from the household with improved types of cook fuel (Table 1).

**Table 1:** Descriptive statistics of infant mortality outcome by study characteristics

Characteristics	Frequency	Percentage
**Infant Survival Status**		
Death	1952	6.5
Alive	28,106	93.5
**Place of Residence**		
Urban	6069	20.2
Rural	23,989	79.8
**Marital status**		
Single	1146	3.8
Married	17,696	58.9
Living with partner	6215	20.7
Widowed	2467	8.2
Divorced/separated	2534	8.4
**Maternal Education**		
No formal education	6778	22.5
Primary	20,409	67.9
Secondary and over	2871	9.6
**Employment status**		
Employed/ self-employed ^1^	28,546	95.0
Unemployed ^2^	1512	5.0
**Household Wealth Index**		
Low	12,610	42.0
Middle	6008	20.0
High	11,440	38.1
**Maternal age at first birth**		
Below 20 year old	9770	32.5
20–34 years old	20,217	67.3
35 years old and over	71	0.2
**Sex of child**		
Male	15,216	50.6
Female	14,842	49.4
**Birth order**		
1 or 2 birth	15,532	51.7
3 or 4 birth	8601	28.6
5 births and over	5925	19.7
**Birth interval**		
Less 24 months	5479	18.2
24 months and over	15,720	52.3
First births	8859	29.5
Birth interval	5479	18.2
**Children ever born**		
1–3 children	9701	32.3
4–6 children	12,698	42.2
Over 6 children	7659	25.5
**Breastfeeding**		
Yes	11,862	39.5
No	18,196	60.5
**Source of drinking water**		
Improved	11,802	39.3
Not improved	18,256	60.7
**Type of toilet facility**		
Improved ^**1**^	28,638	95.3
Not improved ^**2**^	1420	4.7
**Type of cooking**		
Improved	4146	13.8
Not improved	25,912	86.2

*Concerning the toilets, we used two categories of toilet facility*

^***1***^*Babies’s stools are considered to be disposed of safely if the child used a toilet or latrine, if the faecal matter was put or rinsed into a toilet or latrine, or if it was buried*

^***2***^*Facilities that would be considered improved if they were not shared by two or more households. Also, those who had no toilet facility*

*Regarding the type of cooking, two categories that include improved and non-improved were done:*

^***1***^*Improved cooking consisted of those who used gas or biogas, kerosene, electrify or charcoal*

^***2***^*Non-improved include the babies from the families which utilised wood, straw, shrubs, grass, agricultural crop, no food cooked in household*

*In terms of the mother's employment, we divided it into two categories: (1) employed and self-employed; (2) unemployed*

^*1*^* Employed and self-employed are those who have generating income on regular basis*

^*2*^* Unemployed women consist of those who have no generating income on regular basis*

### Bivariate logistic regression for the associated factors of infant mortality

The results showed that place of residence (${x}^{2}=24.1, p<001$), marital status (${x}^{2}=34.2, p<001$), maternal education (${x}^{2}=95, p<001$), wealth index (${x}^{2}=42.2, p<001$), mother’s age at first birth (${x}^{2}=39.7, p<001$), sex of child (${x}^{2}=16.9, p<001$), birth interval (${x}^{2}=249.6, p<001$), children ever born (${x}^{2}=325.9, p<001$), breastfeeding (${x}^{2}=79.6, p<001$), source of drinking water (${x}^{2}=11.4, p<001$), type of toilet facility (${x}^{2}=15.6, p<001$), and type of cook fuel (${x}^{2}=29.7, p<001$) were significantly associated with infant mortality. Infant deaths were relatively higher in rural 6.8% than in urban areas (5.1%). The divorced mothers had a higher rate of infant mortality (8.2%) than other categories of mother’s marital status. Mothers with no formal education reported the highest level of infant mortality (8.6%) when compared to other categories of education. Those with low wealth index reported the highest number of deaths of their infants with 7.4% in relation compared to 6.7% and 5.4% of infant deaths distributed to middle and high wealth index respectively. Mothers who had their first birth below 20 years registered more deaths with 7.8% compared to 5.9% and 4.2% of infant deaths were registered with mothers aged 20–34 years and 35 years or over respectively. Infant mortality was highly prevalent in male children (7.1%). The preceding birth interval of fewer than 24 months was associated with higher risks of infant deaths with 10.8% than those with an interval of 24 months and above with 4.7%. It was observed that mothers with above 6 children experienced the highest number of deaths 10.3% while 3.5% and 6.5% of infant deaths observed to mothers had between one child or 3 children and 4 children or 6 children respectively. Infant deaths were higher associated with households that did not provide breastfeeding to their children (7.5%) than their counterparts. Mothers whose households had the unimproved sources of drinking water had presented more infant deaths with 6.9% than those in households that access the improved sources of drinking water. More deaths were reported in households with no improved toilet facility with 9.0% compared to households with improved toilets facility with 6.4% (Table 2).

**Table 2:** Prevalence of infant mortality by independent variables and associations between independent variables and infant mortality

	**Infants deaths**	**Live births**		
**Characteristics**	*N* (%)	*N* (%)	**Chi-square**	***p*-value**
**Place of residence**				
Urban	310(5.1%)	5759(94.9%)	24.1	< 0.001**
Rural	1642(6.8%)	22,347(93.2%)		
**Marital status**				
Single	61(5.3%)	1085(94.7%)	34.2	< 0.001**
Married	1053(6.0%)	16,643(94.0%)		
Living with partner	434(7.0%)	5781(93.0%)		
Widowed	196(7.9%)	2271(92.1%)		
Divorced/separated	208(8.2%)	2326(91.8%)		
**Maternal education**				
No formal education	583(8.6%)	6195(91.4%)	95	< 0.001**
Primary	1269(6.2%)	19,140(93.8%)		
Secondary and over	100(3.5%)	2771(96.5%)		
**Employment status of women**				
Employed/ self-employed ^1^	1873(6.6%)	26,673(93.4%)	4.2	0.40
Unemployed ^2^	79(5.2%)	1433(94.8%)		
**Household Wealth Index**				
Low	934(7.4%)	1176(92.6%)	42.2	< 0.001**
Middle	405(6.7%)	5603(93.3%)		
High	613(5.4%)	10,827(94.6%)		
**Age of mother at first birth**				
Below 20 year old	760(7.8%)	9010(92.2%)	39.7	< 0.001**
20–34 years old	1189(5.9%)	19,028(94.1%)		
35 years old and over	3(4.2%)	68(95.8%)		
**Sex of child**				
Male	1076(7.1%)	14,140(92.9%)	16.9	< 0.001**
Female	876(5.9%)	13,966(94.1%)		
**Birth order**				
1 or 2 birth	999(6.4%)	14,533(93.6%)	229	0.892
3 or 4 birth	562(6.5%)	8039(93.4%)		
5 births and over	391(6.6%)	5534(93.4%)		
**Birth interval**				
Less 24 months	592(10.8%)	4887(89.2%)	249.6	< 0.001**
24 months and over	746(4.7%)	14,974(95.3%)		
First births	614(6.9%)	8245(93.1%)		
**Children ever born**				
1-3children	336(3.5%)	9365(96.5%)	325.9	< 0.001**
4–6 children	830(6.5%)	11,868(93.5%)		
Over 6 children	786(10.3%)	6873(89.7%)		
**Breastfeeding**				
Yes	584(4.9%)	11,278(95.1%)	79.6	< 0.001**
No	1368(7.5%)	16,828(92.5%)		
**Source of drinking water**				
Improved	696(5.9%)	11,106(94.1%)	11.4	< 0.001**
Not improved	1256(6.9%)	17,000(93.1%)		
**Type of toilet Facility**				
Improved	1824(6.4%)	26,814(93.6%)	15.6	< 0.001**
Not improved	128(9.0%)	1292(91.0%)		
**Type of cook fuel**				
Improved	189(4.6%)	3957(95.4%)	29.7	< 0.001**
Not improved	1763(6.8%)	24,149(93.2%)		

^**^: Statistical significance at p < *0.001*

### Multiple logistic model analysis of factors associated with infant mortality

Multiple logistic models showed that marital status, maternal education, wealth index, sex of child, birth order, birth interval, children ever born, breastfeeding status and type of toilet facility were the significant factors of infant mortality. The infants whose mothers who were widowed or separated from their partners [aOR = 0.7;95% CI (0.51–0.91), *p* = 0.028] had less odds to die than those whose mothers were single. The infants whose mothers studied secondary and over had lower odds to die [aOR = 0.66, 95% CI (0.52–0.85), *p* < 0.001] than the infants whose mothers had no formal education. The births from households with a high wealth index had 0.8 lower risk of dying [aOR = 0.8, 95% CI (0.7–0.9), *p* < 0.001] than those births born from households with a low wealth index. Female infants had lower likelihoods of dying [aOR = 0.8; 95%CI (0.73–0.88), *p* < 0.001] when compared to male infants. The infants born to mothers who had 5^th^ born or more were 0.67 times [aOR = 0.67; 95% CI (0.57–0.79), *p* < 0.001] less likely to die compared to infants born to mothers who had less than 3 children. The infants from mothers whose preceding birth interval was 24 months and above [aOR = 0.45; 95% CI (0.4- 0.51), *p* < 001] and those who had first births [aOR = 0.86; 95% CI (0.74–0.99), *p* = 0.042] had lower odds of dying than those from the mothers with preceding birth intervals of less than 24 months respectively. The infants who were not breastfeed were 1.45 times [aOR = 1.45; 95% CI (1.31–1.61), *p* < 0.001] more likely to die when compared to those who were breastfed. The infants born in families with unimproved toilet facility were 1.34 times more prone to die than those who from the families with improved toilet facility [aOR = 1.34, 95% CI (1.1–1.63), *p* = 0.003] (Table 3).

**Table 3:** Multiple logistic model analysis of factors associated with infant mortality in Rwanda

	**95% Confidence interval**			
	**aOR**	**Lower**	**Upper**	***p*-value**
**Place of Residence**				
Urban	1			
Rural	1.10	0.94	1.29	0.243
**Marital status**				
Single	1			
Married	0.62	0.46	0.82	< 0.001**
Living with partner	0.83	0.62	1.11	0.202
Widowed	0.70	0.51	0.96	0.028*
Divorced/separated	0.88	0.64	1.20	0.408
**Maternal Education**				
No formal education	1			
Primary	0.91	0.82	1.02	0.093
Secondary and over	0.66	0.52	0.85	< 0.001**
**Employment status**				
Employed/ self-employed ^1^	1			
Unemployed ^2^	0.87	0.69	1.11	0.268
**Wealth Index**				
Low	1			
Middle	0.89	0.79	1.01	0.076
High	0.8	0.7	0.9	< 0.001**
**Age of mother at first birth**				
Below 20 year old	1			
20–34 years old	0.91	0.83	1.01	0.066
35 years old and over	1.16	0.36	3.74	0.802
**Sex of child**				
Male	1			
Female	0.8	0.73	0.88	< 0.001**
**Birth order**				
1 or 2 birth	1			
3 or 4 birth	0.93	0.81	1.07	0.299
5 births and over	0.67	0.57	0.79	< 0.001**
**Birth interval**				
Less 24 months	1			
24 months and over	0.45	0.40	0.51	< 0.001**
First births	0.86	0.74	0.99	0.042*
**Children ever born**				
1-3children	1			
4–6 children	2.34	2.02	2.70	< 0.001**
Over 6 children	4.15	3.53	4.88	< 0.001**
**Breastfeeding**				
Yes	1			
No	1.45	1.31	1.61	< 0.001**
**Source of drinking water**				
Improved	1			
Not improved	1.04	0.94	1.16	0.433
**Type of toilet facility**				
Improved	1			
Not improved	1.34	1.10	1.63	0.003*
**Type of cook fuel**				
Improved	1			
Not improved	0.98	0.80	1.21	0.862

***aOR**** Adjusted odds ratio, ****CI*** Confidence intervals, *: statistical significance levels at 0.05, **:* high statistical significance level at 0.001*

### Importance features selection

**Fig. 2:**
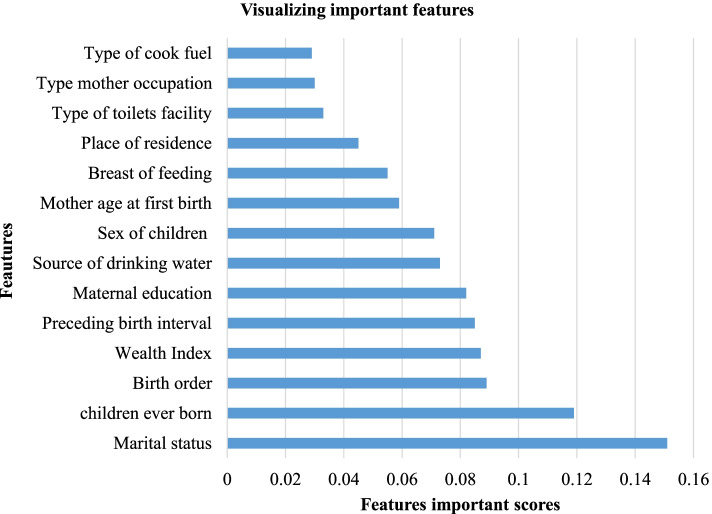
Important features selected for ML

A Random Forest classifier was used to identify important features that are associated with infant mortality as it is shown in Fig. 2. It indicated the top 10 best features that contribute to infant mortality were marital status, children ever born, birth order, wealth index, preceding birth, maternal education, source of drinking water, sex of the child, mother age at first birth and breastfeeding.

### Predicting infant mortality

The ML methods approach models namely Logistic Regression, Random Forests, Decision Tree, and the Support Vector Machine classifiers were applied to build a predictive model of IM. All predictive models of IM were trained on training data of 80% and tested on a test dataset of 20%. The performance predictive models were evaluated and compared using evaluation metrics namely Confusion matrix, Accuracy, Precision, Recall and F1 score, and Area Under receiver operating characteristics AUROC. Our results showed that the logistic regression models predicted correctly 3442 infants died before completing their first year while 3472 infants were still alive. It has wrongly predicted 2114 births died before completing their first years and 2215 births were still alive. Our findings found that the logistic regression model has generally predicted IM at 61.5% of accuracy with recall (61.1%), precision (62.2%), F1 score (61.6%), and AUROC (61.5%). The random forest model was predicted correctly 4283 infants died and 5194 infants as alive. It has wrongly predicted 1273 infants as died and 493 births as still alive. It was found that the random forest model has generally predicted infant deaths correctly at 84.2% of accuracy with recall (91.3%), precision (80.3%), F1 score (85.4%), and AUROC (84.2%). We found that the decision tree model was predicted correctly 4184 infants died and 5151 infants were alive. It has wrongly predicted 1372 births as died and 536 births as still alive. It was generally predicted infant death correctly at 83%) accuracy with recall (91%), precision (79%), F1 score (84.7%), and AUROC (83%). It was found that the Support Vector Machine model was predicted correctly 3454 infants died and 4262 infants were alive. It has wrongly predicted 2102 births as died and 1425 births as still alive. It was generally predicted infant death correctly at 68.6% of accuracy with recall (75%), precision (67%), F1 score (70.7%), and AUROC (68.6%). Based on the predictive model of performance results above, Random Forest was the best predictive model of Infant mortality compared to other models applied in this study (Table 4).

**Table 4:** Predictive models of performance of Infant mortality as evaluated on the test data

			**Predictive Models**							
**Evaluation Matrix**			**Logistic Regression**		**Random Forest**		**Decision Tree**		**Support Vector Machine**	
**Confusion matrix**			**Predicted**		**Predicted**		**Predicted**		**Predicted**	
			**Dead**	**Alive**	**Dead**	**Alive**	**Dead**	**Alive**	**Dead**	**Alive**
	**Observed**	**Dead**	3442	2114	4283	1273	4184	1372	3454	2102
		**Alive**	2215	3472	493	5194	536	5151	1425	4262
			**%**		**%**		**%**		**%**	
**Accuracy**			61.5		84.3		83		68.6	
**Recall**			61.1		91.3		91		75	
**Precision**			62.2		80.3		79		67	
**F1 score**			61.6		85.5		84.7		70.7	
**AUROC**			61.5		84.2		83		68.6	

***AUROC**** Area under the Receiver Operating Characteristics*

## Discussion

This study described the application of ML methods in predicting the infant mortality in Rwanda. This study shows that ML methods predict the factors associated with infant mortality is better than the logistic regression models or traditional methods. This result is not surprising, since ML methods are documented to outperform logistic methods in several fields of medicine. These findings were relevant to the findings of prior studies [37–40]. By using Random Forest classifier methods, the findings revealed that province of residence, household wealth index, sex of children, maternal education, source of drinking water, maternal age at first birth, birth order, marital status, child twin, breastfeeding status, and form of residence and number of children ever born were all important risk factors associated with infant mortality. These results collaborated with findings of a prior study that employed predictive models and reported that household size, maternal education, breastfeeding, source of water, bod mass index of mother, birth intervals, antenatal care service and birth order as the predictors of infant mortality [26, 41].

Regarding the predictive analysis, the prediction accuracies and AUC statistics revealed the highest for the Random Forest model. Our results confirmed a higher predictive power compared to the other ML models included in this study. The random forest model was predicted correctly for 4160 infants as dead and 1396 infants as alive. It has wrongly predicted 815 infants as died and 1,396 births as still alive. It was found that the random forest model has generally predicted infant deaths correctly at 80.3% of accuracy with recall: 85.6%, precision: 77.7%, F1 score: 81.5%, and AUROC: 80.3%. These results are in congruence with the previous studies [38, 42], however, a few study contradicted this by indicating a low predictive accuracy [4]. The decision tree model was predicted correctly 4134 infants died and 4833 infants as alive. It has wrongly predicted 854 births as died and 1422 births as still alive. It was generally predicted infant death correctly at 79.8% of accuracy with recall: 85%, precision: 77.3%, F1 score: 80.9%, and AUROC: 79.7%. These findings were similar to the earlier studies [38, 43, 44]. Support Vector Machine model was predicted correctly 3528 infants as died and 3918 infants as alive. It has wrongly predicted 1,769 births as died and 2,082 births as still alive. It was generally predicted infant death correctly at 66.2% of accuracy with recall: 68.9%, precision: 65.9%, F1 score: 67.4%, and AUROC: 66.2%. These results were analogous to prior studies [39, 45].

Logistic regression models predicted correctly 3,710 infants were died before completing their first years while 3,369 infants were still alive. It has wrongly predicted 2318 births will die before completing the first years and 1846 births were still alive. In congruence with prior studies [4, 13, 46], our results from the logistic regression model have generally predicted IM at 62.3% of accuracy with recall: 59.24%, precision: 64.6%, F1 score: 61.8%, and AUROC: 63%. Based on the performance of our predictive model, Random Forest was the best predictive model of infant mortality compared to findings from the other models since it had the highest scores of different evaluation metrics used in this study. In a similar vein with earlier studies [4, 13, 20], our results revealed that ML methods or deep learning models are better than the traditional analytical approaches. Hence, our predictive analyses revealed that the prediction accuracies and AUC statistics are the highest for the RF model. This RF model also agreed a higher predictive power than the other models used in this study. In incongruity with previous studies conducted by Shukla [4], our findings from the RF models demonstrated that marital status, children ever born, birth order and wealth index were the 4 top associated predictors of infant mortality. In concur with the other study conducted by Bitew 2020 [26], our study revealed that birth order, wealth index, source of drinking water, sex of child and breastfeed are among the top predictors associated with infant mortality. Therefore, our overall results confirmed that ML methods significantly provide better discrimination than the traditional models in assessing the factors associated with IM. These results are in a similar vein with past studies [4, 29] that documented that the ML methods are more appropriate methods to determine factors associated with infant mortality and it presents goodness of fit in most critical groups [40, 46, 47].

This research had numerous prominent strengths. The first strength is that the RDHS 2014/2015 used and standardized tools for interviewing participants. Second, the research data about infant mortality that we used were obviously verified through records and recall bias was prevented. However, several limitations also warrant discussion. First, it was limited to a study design that did not actually explore for modifiable factors of IM. Second, the study was also limited to the bias of selection process that resulted in the underestimation of the association between infant mortality. Indeed, the study was limited to lack of qualitative aspects data from the participants for exploring some factors and to triangulate the findings of the quantitative methods used. Forth, as ML method is novel and was not employed in SSA countries, we did not have enough scientific evidence to compare with other SSA countries where Rwanda is located. Third, eventually, unlike the multivariate logistic regression models considered as the traditional logistic regression analyses, the results from the ML appear not to be interpretable due to not having regression coefficients. ML methods predict the factors based on the level of importance of their role in assessing the magnitude of mortality within this work. Therefore, existing epidemiological literature from the studies used multivariate logistic regression models that measured the directions of the associated factors of IM. Fifth, our algorithm of ML was designed in a retrospective manner after the measurements for a certain period are taken since we employed secondary data analysis, so, this requires some adaptations for real-time applications which possibly lead to a drop in the performance. Sixth, our study was limited to the study design, the cross-sectional design that could only infer associations and not causality. This allowed as recommending further research on the longitudinal study design to assess the risk factors of infant mortality using the ML methods.

## Conclusion

In developing a predictive model, ML approaches are strong and can be used to classify certain secret knowledge that could not be detected by conventional statistical methods. Thus, ML methods revealed that marital status, children ever born, birth order and wealth index are the top predictors of IM. Based to this, ML techniques can improve the accuracy of the algorithm and use training data for the training model and use unseen test data to make predictions. The general goals of this study were to apply methods of ML, namely logistic regression, random forest, decision tree, and support vector machine in the analysis of infant mortality in Rwanda. As an example of conventional statistical approaches that can be used to construct predictive models, logistic regression was used. The residence, wealth index, sex of children, maternal education, source of drinking water, age of the mother at first birth, birth order, marital status, child twin, breastfeeding, place of residence, and the number of children ever born were the main factors by applying random forest methods to select best features associated with infant mortality. ML approaches have high output accuracy compared to conventional statistical methods. Among the four ML algorithms used in this study, the random forest was classified as the best classifier to be used for the predictive model of infant mortality in Rwanda compared to other ML models used in this study. Our approaches, ML methods, are recommended to be adapted to tackle other health outcomes such as survival very preterm, neonatal mortality, stunting, and low birth weight infants that remain public health concerns in Rwanda.

## Data Availability

Underlying data for “Application of ML methods in the analysis of infant mortality in Rwanda: Analysis of Rwanda Demographic Health Survey 2014–15 Dataset” was owned by the DHS program that can be obtained from https://dhsprogram.com/methodology/survey/survey-display-468.cfm. The electronic data is available from the DHS program under its terms of use. Before downloading the data, the main author of this study registered as DHS user for reasons laid out on the DHS program website and dataset access was only granted for legitimate purpose of this research. The name of the main author of this study is Emmanuel Mfateneza.
